# Ultrastructure and regulation of color change in blue spots of leopard coral trout *Plectropomus leopardus*

**DOI:** 10.3389/fendo.2022.984081

**Published:** 2022-10-20

**Authors:** Nannan Zhao, Xiaoyu Ge, Ke Jiang, Jing Huang, Ke Wei, Chao Sun, Shi Xi Chen

**Affiliations:** ^1^ State Key Laboratory of Marine Environmental Science, College of Ocean and Earth Sciences, Xiamen University, Xiamen, Fujian, China; ^2^ State-Province Joint Engineering Laboratory of Marine Bioproducts and Technology, Xiamen University, Xiamen, Fujian, China

**Keywords:** leopard coral trout, blue spots, forskolin, color change, sympathetic nerves, norepinephrine

## Abstract

The leopard coral trout generally exhibited numerous round, minute blue spots covering its head (about the size of nostril) and body (except ventral side). This is a characteristic that distinguishes them from similar species. Recently, however, we found the leopard coral trout with black spots. Here, the distribution and ultrastructure of chromatophores in the blue and black spots were investigated with light and transmission electron microscopies. The results showed that in the blue spots, two types of chromatophores are present in the dermis, with the light-reflecting iridophores located in the upper layer and the aggregated light-absorbing melanophores in the lower layer. Black spots have a similar chromatophore composition, except that the melanosomes within the melanophores disperse their dendritic processes to encircle the iridophores. Interestingly, after the treatment of forskolin, a potent adenylate cyclase activator, the blue spots on the body surface turned black. On the other hand, using the skin preparations *in vitro*, the electrical stimulation and norepinephrine treatment returned the spots to blue color again, indicating the sympathetic nerves were involved in regulating the coloration of blue spots. Taken together, our results revealed that the blue spots of the leopard coral trout can change color to black and vice versa, resulting from the differences in the distribution of melanosomes, which enriches our understanding of the body color and color changes of fishes.

## Introduction

The leopard coral trout, *Plectropomus leopardus* (also known as the coral trout or spotted coral grouper), is a representative member of the genus *Plectropomus* (1). It is a carnivorous coral reef fish widely distributed in tropical and subtropical waters from Western Pacific to East Africa and the Red Sea (1, 2). They are easily distinguished from similar species by their characteristic blue spots (3), which are round and minute (approximately the size of the nostril), covering the entire body except for the ventral side (4). Blue coloration have been found in a variety of fish including zebrafish *Danio rerio* (5), blue damselfish *Chrysiptera cyanea* (6), common surgeonfish *Paracanthurus hepatus* (7), Siamese fighting fish *Betta splendens* (8), male guppies *Poecilia reticulata* (9), the mandarin fish *Synchiropus splendidus* and the psychedelic fish *S. picturatus* (10), etc. It was concluded that blue coloration are achieved by a great variety of mechanisms (11). Usually, blues are structural colors whose wavelengths are reflected as a result of optical interference by nanoscale structures in or on an animal’s integument (11–13). In vertebrates, the iridophore is a chromatophore that contains crystalline structures (colour-producing nanostructures) that give rise to blue colouration (13–15). For example, in the stripe regions of the zebrafish trunk, type S iridophores were found (16); multilayered thin-film interference occurring in stacks of crystalline reflecting platelets within iridophores provides the basis for the expression of their blue iridescence (12). In addition, the first and only known cyanophores (ture blue chromatophores) have been discovered in the ectoderm of two callionymid species, the mandarin fish and the psychedelic fish (11). Cyanophores are dendritic and contained many blue pigmentary organelles, cyanosomes, which generate bluish coloration by their absorption of light (10). So far, the mechanism underlying the blue coloration of blue spots in leopard coral trout is unclear and yet to be explored.

In addition to those with common blue spots, the leopard coral trout with black spots have also been observed. Different from the blue color, the black body color is commonly produced by eumelanin within melanophores (17–19). It has been reported in several species that fish from different geographical populations exhibit distinct body colors (4, 20, 21). This hints at the possibility that the black-spotted and blue-spotted leopard coral trout might belong to two geographical populations of the same species, differing in the phenotype of coloration. In addition to this polymorphism, many fish species can modify the body brightness or hues in response to environmental factors, known as polyphenism (22). These changes are classified as morphological and physiological (23–25). The former can be attributed mainly to increases or decreases in the actual number of chromatophores or the amount of pigmentary material inside (26). On the other hand, the physiological color changes are attributed to the rapid migration of chromatosomes centripetally or centrifugally or changes in the arrangement of high refractive index materials in the cytoplasm (27, 28). For example, in response to various stimuli, the cyanophores in the bluish skin of the mandarin fish and the psychedelic fish responded by the aggregation or dispersion of cyanosomes (10), and in the bluish skin of blue damselfish, the distance between the reflecting platelets in the iridophores changed (29). Moreover, interactions between different chromatophores layers also play an important role in the physiological color changes (30). Body color polyphenism has also been reported in many other fish species, including common surgeonfish (7), large yellow croaker *Larimichthys crocea* (31), paradise whiptail *Pentapodus paradiseus* (32) and Domino Damsel *Dascyllus trimaculatus* (33), etc. This raises another possibility that the black and blue spots of leopard coral trout might be caused by changes in body color.

In the present study, the chromatophores (ultra)structure of the blue and black spots of the leopard coral trout were examined by light and electron microscopies. The effect of forskolin, an adenylate cyclase (AC) activator (34), on the spots color was investigated. Furthermore, the possible involvement of the sympathetic nervous system in controlling the coloration of spots was studied *in vitro*.

## Material and method

### Experimental fish

The leopard coral trout were purchased from Xiao Deng Fisheries Technology Co., LTD and Xia Shang Aquatic Market (Xiamen, Fujian province), with body lengths of 30 - 35 cm and weights of 400 - 450 g. Fish were reared in indoor plastic tanks with seawater at salinity 30 and temperature 25°C for about one week before use.

### Sampling and light microscopy

The leopard coral trout were anesthetized with 100 mg/L Ethyl 3-aminobenzoate methanesulfonate (MS222, Sigma Aldrich, CAS: 886-86-2) and then placed on ice for sampling. Skin with spots were dissected carefully for light microscopy and transmission electron microscopy (TEM) analyses. For the light microscopy analysis, the skin was washed three times with 0.1 M phosphate-buffered saline (PBS, dissolve the 8 g NaCl, 0.2 g KCl, 3.63 g Na2HPO4-12H2O and 0.24 g KH2PO4 to 1L) (pH 7.4), fixed in 4% paraformaldehyde for 24 h at 4 °C, and then photographed using a light microscope (Leica, DM2500 LED) equipped with a Leica DFC7000 T camera.

### Semi-thin sections and TEM

For the TEM analysis, the skin samples were washed three times in PBS and then fixed in 2.5% glutaric dialdehyde in 0.2 M phosphate buffer at 4°C overnight. After being post-fixed in 2% osmium tetraoxide in 0.2 M phosphate buffer for 2 h at 4°C, the skin samples were washed three times in distilled water, dehydrated in a graded series of acetone solutions (50, 70, 90, and 100% for 15 min at 4°C and twice each step, soaked in acetone: resin mixture (3:1) twice for 30 minutes, and immediately embedded in a Spurr resin (SPI, West Chester, USA).

To localize blue or black spots, semi-thin sections (0.8 μm) were made vertically to the plane of the skin with a glass knife on an ultramicrotome (Leica EM UC7). The sections were stained with 1% toluidine blue (in borate buffer) for 3 min at 60°C. Images were captured with a light microscope (Leica, DM2500 LED) equipped with a Leica DFC7000 T camera. Regions of interest for the further TEM analysis were selected based on the preliminary analysis of the semi-thin sections. Ultrathin (65 nm) sections were cut vertically to the plane of the skin using an ultramicrotome with a diamond knife, and then mounted on formvar-coated copper grids (Electron Microscopy Sciences, Hatfield, PA). At last, the sections were stained with uranyl acetate and lead citrate, and observed under an electron microscope (Philips CM100).

### Effect of forskolin on spots color change *in vivo*

Forskolin (Selleck Chemicals, Houston, TX, USA), an AC stimulating agent, was first dissolved in DMSO (Solarbio, Beijing, China) at a concentration of 10 mM, and then diluted in PBS to a final concentration of 100 μM, which was applied to the blue spots of the body surface, and the color change of spots was recorded with SONY camera (DSC-HX300). The experiment was repeated three times on three different fish. The changes in diameter of spots in the color change process were assessed by Nano Measurer 1.2 (n=30 spots). We used online tools Image Color Detection to extract the hue of the blue (n=6 spots) and black spots (n=6 spots) and obtain the color code. Then, we obtained the corresponding HSL and RGB values with the help of the color code converter in Rappidtables.

### Acquisition of excised skin pieces

The leopard coral trout was anesthetized with 100 mg/L MS222 and sampled at room temperature. The skin with spots was dissected with a sterilized scalpel. The skin was first kept in the original position and the color of the spots was photographed using a SONY camera (DSC-HX300). Then, the skin was sliced in half and immediately immersed in a physiological saline solution of the following composition (mM): NaCl 125.3, KC1 2.7, CaC1_2_ 1.8, MgC1_2_ 1.8, D-glucose 5.6, and Tris-HC1 buffer 5.0 (pH 7.2) for the electrical stimulation experiment and *in vitro* incubation.

### Electrical stimulation treatments

A JL-A electronic stimulator (Shanghai, China) was used to perform the electrical stimulation. A unidirectional square pulse of constant pulse width (0.6 ms) with no interphase gap, 40 Hz in stimulation frequencies and 50 V in strength were applied for 0.25 s. The electrode was placed on a portion of the excised skin pieces and the rest of the skin was used as a control. Images were taken using a SONY camera (DSC-HX300) before and after the electrical stimulation and 1 min after the cessation of stimulation. The experiment was repeated three times on three different fish.

### Effects of NE and forskolin on spots color change *in vitro*

Norepinephrine (NE) (Macklin, N814761, CAS: 51-41-2) was dissolved into 10 mL PBS to obtain a stock solution at a concentration of 1 mM and then diluted with PBS to a final concentration of 10 μM. NE was applied to one side of the excised skin and the rest of the skin was used as control, treated with PBS. After 5 minutes, 100 μM forskolin was applied to the NE treated side. The color changes of the spots were observed and photographed using a SONY camera (DSC-HX300).

## Results

### The blue color of spots is due to the light reflection of iridophores

**Figure 1:**
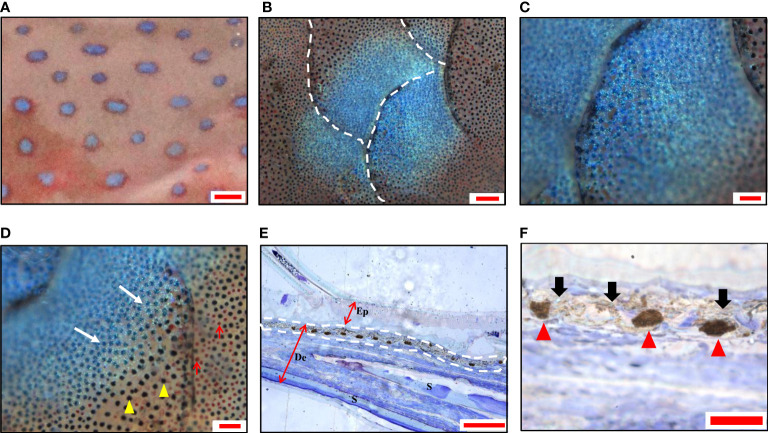
Morphology and chromatophores distribution of blue spots in leopard coral trout *Plectropomus leopardus*. **(A)** A magnified image of the skin shows blue spots with irregular shapes. **(B)** A panoramic image shows blue spots distributed over multiple scales under a light microscope. **(C)** The center of the spot is dark blue, and **(D)** the surrounding blue becomes lighter. Melanophores (yellow arrowheads); Erythrophores (red arrows). **(E)** A representative image of semi-thin sections (toluidine blue staining), shows continuously aggregated melanophores in the dermis. The white dashed line indicates the position of chromatophores. Ep, epidermis; De, dermis; S, scale. **(F)** The magnification of the partial image in **(E)** shows aggregated melanophores (red arrowhead) and iridophores (black arrow). Scale bars: 2 mm **(A)**; 200 μm **(B)**; 100 μm **(C–E)**; 25 μm **(F)**.

The blue spots were about 1-2 mm in diameter and scattered on the skin (**Figure 1A**). Under light microscopy, the blue spots showed rounded shapes and distributed across multiple scales (**Figure 1B**), with darken blue in the center (**Figure 1C**) and lighter blue around (**Figure 1D**). The semi-thin sections showed continuous clusters of aggregated melanophores in the dermis, with the iridophores above them (**Figures 1E, F**). No chromatophores were observed in the epidermis.

**Figure 2:**
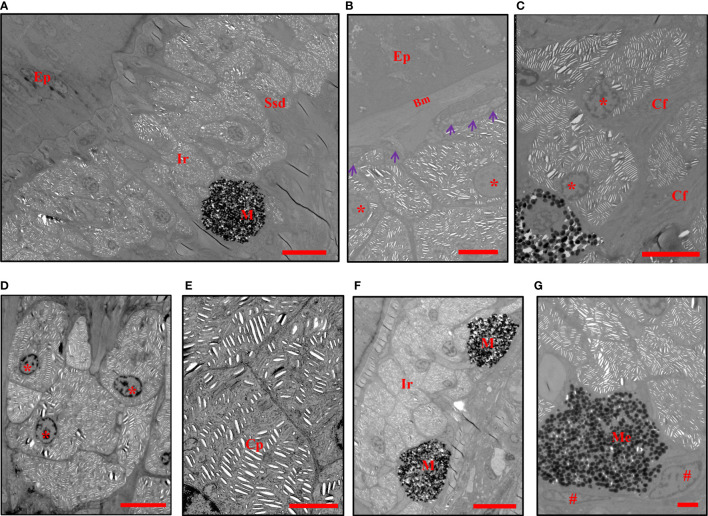
TEMs show the chromatophores in blue spots. **(A)** The iridophores and melanophores are located in the stratum spongiosum of the dermis. **(B)** The iridophores are located below the basal lamella without direct contact (purple arrowheads). **(C, D)** The arrangement and shape of iridophores. **(E)** Crystalline platelets within melanophores. **(F)** The melanosomes are totally aggregated in the perikarya of melanophores. **(G)** Melanophores are in direct contact with the lower surface of iridophores. Ep, epidermis, Ssd, stratum spongiosum of the dermis, Bm, basement membrane, Cf, collagen fibers, M, melanophores, Me, melanosomes, Ir, iridophores, Cp, crystalline platelets. The nuclei of iridophores are indicated with an asterisk *, and those of melanophores are indicated with a pound sign #. Scale bars: 10 μm **(A, F)**; 5 μm **(B–D)**; 2 μm **(E, G)**.

The TEM analysis confirmed the presence of two types of chromatophore (iridophores and melanophores) in the center of the blue spots and positioned in the stratum spongiosum of the dermis (**Figure 2A**), while several isolated chromatophores were observed in a deeper layer (data not shown). The non-dendritic iridophores were arranged in multiple layers (**Figure 2A**), located below the basal lamella but not directly in contact (**Figure 2B**), and were either closely connected or separated by collagen fibers (**Figure 2C**). The iridophores were round, oval, or polygonal in shape and contained a rounded or elliptical nucleus which was generally found near the base of the cell (**Figure 2D**). In iridophores, there were no regular patterns in the distribution and the orientation of stacks of parallel lacunae. These lacunae were usually observed as empty spaces, but occasionally thin platelets were seen filling these spaces or parts of them (**Figure 2E**). The deeper dermal chromatophores were melanophores, the body of which were in intimate contact with the lower surface of iridophores (**Figures 2F, G**). The melanosomes in melanophores were aggregated and close to the nucleus (**Figure 2G**). These results indicated that the overlying reflective iridophores contribute to the observed blue color of spots.

### The black color of spots is caused by the light absorption of melanosomes within melanophores

**Figure 3:**
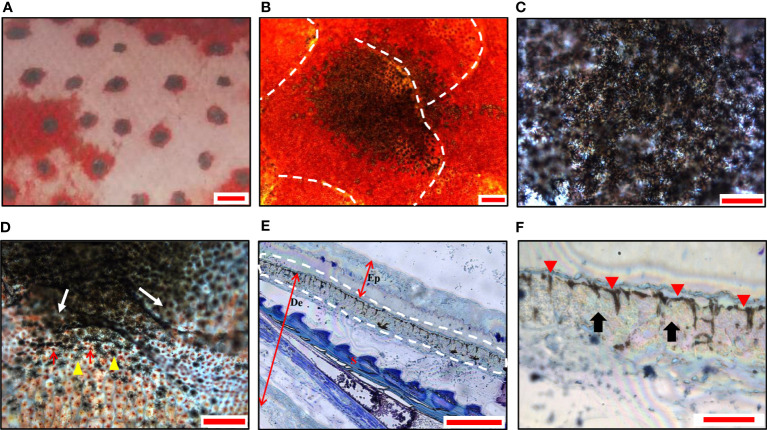
Morphology and chromatophores distribution of black spots in leopard coral trout. **(A)** A magnified image of the skin shows black spots with irregular shaped. **(B)** A panoramic image shows black spots distributed over multiple scales under a light microscope. **(C)** The center of the spot is black. **(D)** The edges of the black spots (white arrows). Melanophores (yellow arrowheads); Erythrophores (red arrows). **(E)** A representative image of semi-thin sections (toluidine blue staining), shows continuously dispersed melanophores in the dermis. The white dashed line indicates the chromatophores. Ep, epidermis; De, dermis; S, scale. **(F)** The magnification of the partial image in **(E)** shows dispersed melanophores (red arrowheads) and covered iridophores (black arrows). Scale bars: 2 mm **(A)**; 200 μm **(B)**; 100 μm **(C–E)**; 25 μm **(F)**.

Similar to the blue spots, the black spots on the skin were also 1-2 mm in diameter (**Figure 3A**) and showed similar irregular shapes across multiple scales (**Figure 3B**). Dark black was observed in the center (**Figure 3C**), and gradually replaced by red color toward the edge of the spots (**Figure 3D**). The semi-thin sections revealed a distinct continuous band of chromatophores in the center of the black spots, in which the dendrites of the melanophores were clearly visible, mostly above the iridophores (**Figures 3E, F**). There were no chromatophores in the epidermis.

**Figure 4:**
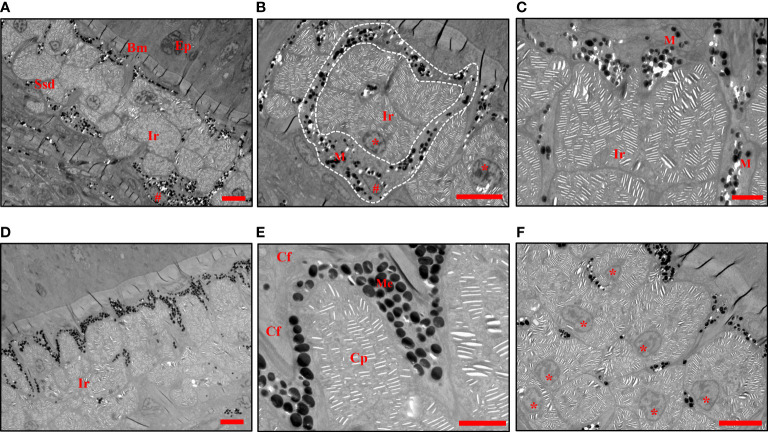
TEMs show the chromatophores in black spots. **(A)** The iridophores and melanophores are located in the stratum spongiosum of the dermis. **(B)** Melanophores containing dispersed melanosomes are seen around the iridophores (white dashed line). **(C)** Branches of melanophores extend their dendrites through the space between the iridophores. **(D)** Melanophores containing dispersed melanosomes are seen cover the iridophores. **(E)** The melanosomes extend up into the space between the uppermost iridophore and the subepidermal collagenous space. **(F)** The arrangement, shape of iridophores and their nuclei. The crystalline platelets are randomly arranged. Ep, epidermis, Ssd, stratum spongiosum of the dermis, Bm, basement membrane, Cf, collagen fibers, M, melanophores, Me, melanosomes, Ir, iridophores, Cp, crystalline platelets. The nuclei of iridophores are indicated with an asterisk *, and those of melanophores are indicated with a pound sign #. Scale bars: 5 μm **(A, B, D, F)**; 2 μm **(C, E)**.

The TEM analysis further verified the morphology of melanophores and iridophores in stratum spongiosum of the dermis at the center of the black spots (**Figure 4A**). Several isolated chromatophores were also noticed in the deeper layer (data not shown). A thick accumulation of non-dendritic iridophores was present in the dermis, which was lined with the layer of melanophores (**Figure 4A**). Melanophores were located below the iridophores but extended their dendrites to encircle iridophores (**Figures 4B, C**). Melanosomes were found dispersed to the distal part, and can extend up into the space between the iridophores and the subepidermal collagenous space (**Figures 4D, E**). The obscured iridophores were round, oval, or polygonal in shape (**Figure 4F**). Each cell contained a round or oval nucleus located in its basal part, from where stacks of parallel lacunae of light-reflecting platelets radiated into the cytoplasm. The direction of every stack was rather random (**Figures 4E, F**). These results indicated that the black color of the spots is due to the light absorption of the melanosomes locating on top of iridophores.

### Dispersion of melanosomes within the melanophores induced the blackening of blue spots

**Figure 5:**
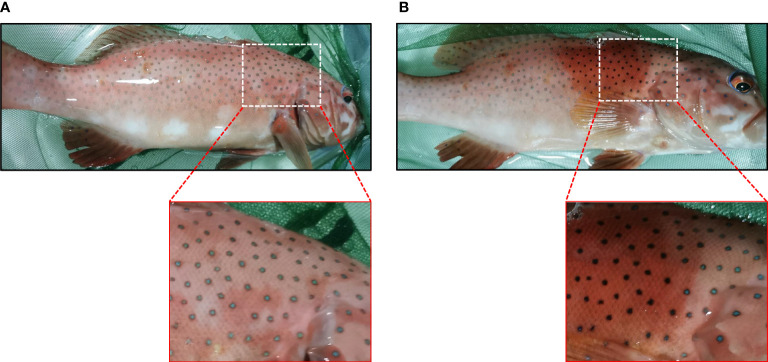
Photographs (taken with a camera) showing the color change of spots. **(A)** Upper panel, blue spots before forskolin treatment. Lower right, the enlargement of the boxed area in the above image. **(B)** Upper panel, 5 min after the treatment of 100 μM forskolin. Lower right, the enlargement of the boxed area in the above image.

Both the blue and black spots were composed of iridophores and melanophores. Therefore, we speculated that the blue and black spots are two body color variations of the same spot, which results from the migration of melanosomes. To confirm this hypothesis, we further examined whether forskolin, which has been reported to induce melanophores dispersion (35), can induce color change of the blue spots. The blue spots treated with 100 μM forskolin turned black within 2 min (**Figures 5A, B**), suggesting that the chromatosomes dispersion contributes to the color change of spots. The diameter of the spot increased to about 1.22 times as it changed from blue to black. The spots vary in hue from the initial 7-13 (R: 23-27; G value: 9-13; B values: 5-7) to 99-119 (R values: 51-89; G value: 86-134; B value: 70-121).

### Sympathetic release of NE causes the spots to manifest blue

**Figure 6:**
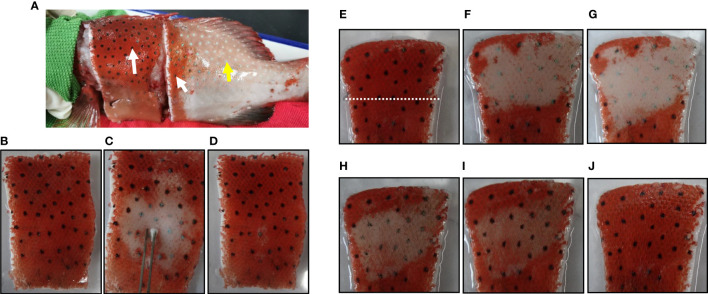
Photographs (taken with a camera) showing the spots on the excised skin and their responses to electrical stimulation, norepinephrine or forskolin. **(A)** Black spots on and around the excised skin (white arrows) and blue spots elsewhere (yellow arrow). **(B)** Equilibrated in physiological saline. **(C)** 15 sec after the electrical stimulation. **(D)** 1 min after the cessation of electrical stimulation. **(E)** Equilibrated in physiological saline. 10 μM NE is applied to the area above the white dashed line and the lower part is used as a control. **(F)** 3 min after the treatment of NE. **(G)** 5 min after the treatment of NE. 100 μM forskolin was applied directly to the area where the color has changed. **(H)** 1 min after the treatment of forskolin. **(I)** 2 min after the treatment of forskolin. **(J)** 10 min after the treatment of forskolin.

To further clarify whether forskolin can induce spots darkening *in vitro*, the skin was excised from an anesthetized leopard coral trout. Surprisingly, once the skin was excised, the blue spots turned black spontaneously and quickly (**Figure 6A**). The rapid darkening of the excised skin was typical of peripheral sympathetic nerve disconnection (23), suggesting a role for sympathetic nerves in innervating spots color change.

Electrical stimulation of nerve fibers in the excised skin induced the spots color to turn blue (**Figures 6B, C**). Once the electrical stimulation was stopped, the blue color of the spots reversed to black within 1 min (**Figure 6D**). Furthermore, after treatment with 10 µM NE for 5 min, the black spots on the excised skin turned blue (**Figures 6E–G**). These results suggested that sympathetic nerves regulated the color change of the spots by releasing neurotransmitter NE. Furtherly, after treatment with 100 μM forskolin for 10 min, the effect of NE was reversed and the spots on the excised skin appear black again (**Figures 6H–J**). These results demonstrated that forskolin could also disperse melanosomes on the excised skin.

## Discussion

In vertebrates, blue coloration is manifested in an array of different fish species (13), shch as the coral reef blue damselfish (6), common surgeonfish (7), striped surgeonfish *Acanthurus lineatus* (36), reef parrotfish *Sparisoma amplum* (37) and five lined snapper *Lutjanus quinquelineatus* (38), etc. The vast majority of the fishes with blue color in their skin have actually no true blue chromatophores (11). The iridophore is a chromatophore containing crystalline structures that give rise to blue coloration by selective light scattering (13–15). For example, the dermis of the blue damselfish contains a single layer of rounded or ellipsoidal iridophores and their multilayered thin-film interference of non-ideal status was responsible for the generation of the cobalt blue color (39). Among other blue fish found in association with coral reefs is the common surgeonfish, whose purity of the blue hue, described as “sky blue” or “cerulean blue”, is rather low in comparison with that in blue damselfish (13). TEM showed round iridophores without dendritic processes were compactly arranged in a double layer in the uppermost part of the dermis (40). In leopard coral trout, triple or quadruple layer of iridophores were observed in the dermis under the blue spots, differing from those of above two blue reef fishes. The iridophores are round, oval, or polygonal in shape, with platelets of random orientation distributed within them, producing a lighter blue tint than sky blue. The variation in the formation of iridophore layers may be a decisive factor in determining the type of blue colors (8), and it is hypothesized that incident light rays are scattered more differentially in the reflecting platelet strata of a multilayer, dual-layer than a monolayer, or simpler light reflecting system results in purer blue coloration (7, 13).

**Figure 7:**
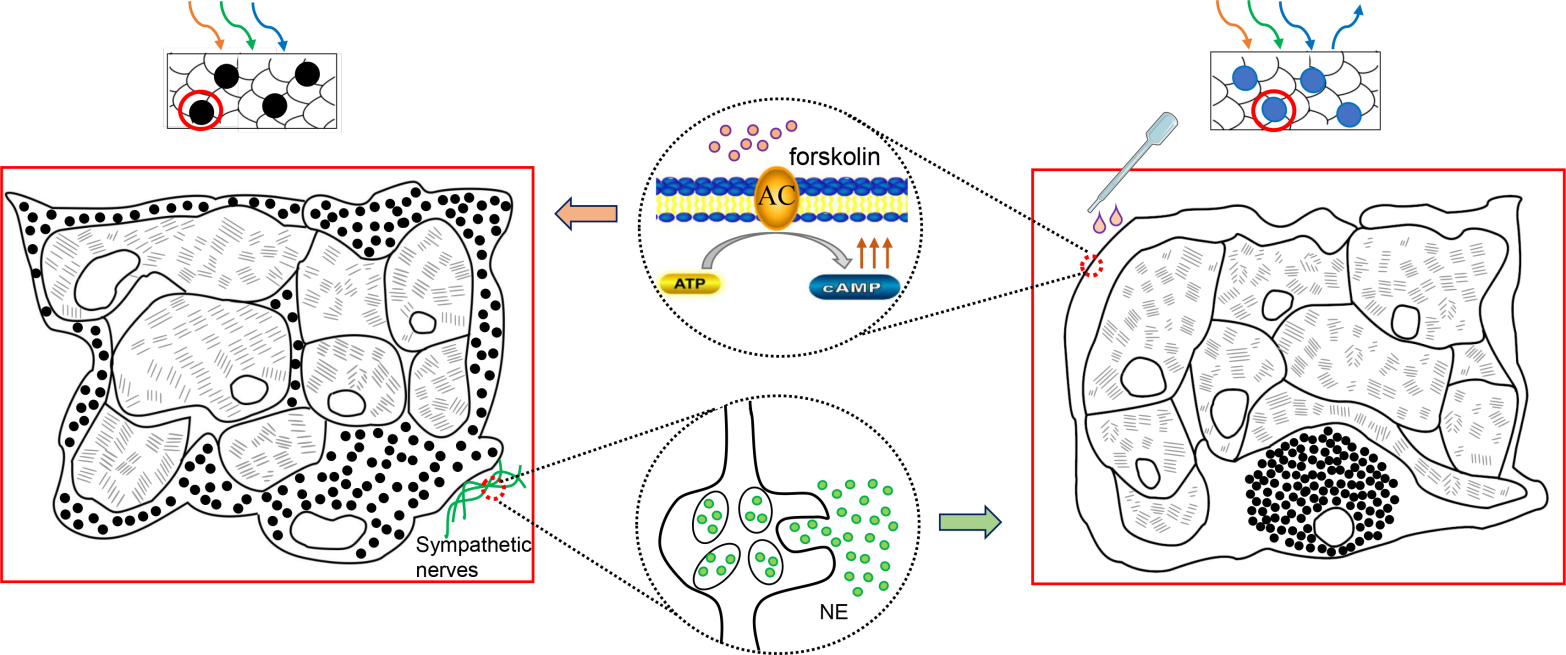
Schematic graph shows color changes between blue and black spots. The sympathetic release of NE drives the aggregation of melanosomes to the perinuclear area. Only the light reflected by iridophores is visible, and the spots appear blue. Forskolin can induce skin darkening by elevating intracellular cAMP. The melanosomes dispersed distally, surround or cover the iridophores and absorb light, giving the spots a black color.

In fish, pigment patterns predominantly result from the positioning of differently colored chromatophores (41). However, the results from present study showed that both black and blue spots were composed of similar melano-iridophore complexes (42). In black spots, melanophores are in a dispersed state, which surround the iridophores and prevent light from reaching reflecting platelets, leading to skin darkening. In blue spots, melanophores are in an aggregated state, only the light reflected by iridophores is visible as remaining wavelength are absorbed by the underlying melanosomes (**Figure 7**). These two structures of chromatophores have been observed in many species that can change their body colors, where the dispersion or aggregation of melanosomes (i.e., obscuring or exposing the iridophores) results in darkening or blanching of the body color (7, 33, 41). For instance, the sky-blue portions of common surgeonfish shows circadian changes, and in the specimen fixed at the daytime, melanophores were found with dispersed melanosomes whose dendritic processes extending into spaces among iridophores. During the night, this portions became paler to a purplish color, where melanosomes were totally aggregated in the perikarya (7). Therefore, it can be inferred that the spots color of leopard coral trout also varies physiologically, either black or blue, depending on the relative position of the melanosomes within melanophores. Subsequently, forskolin was applied on the body surface and it induced a clear darkening of the blue spots, confirming that the blue and black spots are indeed a body color variation (polyphenism) rather than polymorphisms. Meanwhile, forskolin has been reported to directly activate adenylyl cyclase enzyme, that generates cyclic adenosine monophosphate (cAMP) from adenosine triphosphate (ATP), thus, raising intracellular cAMP levels (43, 44). This suggests that elevated intracellular second messenger cAMP mediates the dispersion of melanophores, turning the spots from blue to black (**Figure 7**). The phenomenon has been demonstrated in several species, including spotted triplefin *Grahamina capito* (45), Siamese fighting fish (46), sand goby *Pomatoschistus minutes* (47) and large yellow croaker (31) etc, suggesting that this is a conserved mechanism among species.

The motile activities of chromatophores of teleosts are regulated effectively by the endocrine and/or nervous systems (27, 42, 48, 49). Here, we observed that severing of peripheral nerves in the skin gives rise to a rapid darkening of the spots, which is due to the dispersion of the melanosomes within the melanophores in these areas (50). As observed in minnow *Phoxinus phoxinus*, after severing of the superficial ophthalmic nerve, melanophore dispersion occurred in the corresponding quadrant of the head (51). Similarly, electrical stimulation of the skin preparations of leopard coral trout produced complete melanophore aggregation, thus, reversed the spots color of the area darkened by nerve-section, like that in minnow, indicating that these melanophores are under the control of the nervous system (42).

Furtherly, it has now been established that the nervous control of chromatophores in teleosts is exerted by the sympathetic system (50) and NE, a neurotransmitter of the sympathetic fibers, is believed to be responsible for signal transmission and leads to pigment aggregation (23, 42, 52–59). In our present study, after being treated with NE, the black spots on the excised skin changed to blue color, demonstrating that the color of spots are regulated by sympathetic nerves, and the neurotransmitter NE plays an role in regulating malanophores aggregation to make spots appear blue. Moreover, we found that the effect of NE could be reversed by forskolin. It has generally been accepted that the action mechanisms of catecholamines are cAMP-dependent (42), such as the studies in melanophores from cuckoo wrasse *Labrus ossifagus* and medaka *Oryzias latipes* (54, 60). More specifically, a decrease in intracellular cAMP level induces pigment aggregation in fish chromatophores (61). Thereby, we hypothesize that the NE-induced aggregation of melanophores, which turns the spots from black to blue, may be the result of a decrease in cAMP level.

Many animals change their coloration in response to specific situations and the biological reasons of these color changes are multiple, ranging from thermoregulation, dynamic camouflage to inter- and intraspecific communication (22, 62, 63). Based on previous studies of coral reef fish, we speculate that the blue spots of leopard coral trout may be a form of interspecies communication, as Marshall et al. have describes: “Yellow and blue are the best colours for communication in reef waters as they transmit the furthest and provide the greatest chromatic contrast for a variety of visual systems” (64). In addition, we speculate that the color change of spots from blue to black may be a predation strategy, also have been described by Marshall et al.: “One driver for colour change in reef fishes is to facilitate predation” (64) and observed in the Nassau groupers *Epinephelus striatus* (65). Linking the mechanism (proximate causes) underlying reversible color changes to their biological functions (ultimate causes) is of great importance, especially in the field of evolutionary biology (22). But in any case, the spots of leopard coral trout provide us an excellent material for studying the mechanisms of rapid color changes and a interesting system for further physiological studies from a cell biological perspective.

## Data availability statement

The raw data supporting the conclusions of this article will be made available by the authors, without undue reservation.

## Ethics statement

The animal study was reviewed and approved by Laboratory Animal Management and Ethics Committee of Xiamen University; Xiamen University.

## Author contributions

NZ and SC designed the study. NZ, XG, KJ, KW and CS collected the data. NZ and SC analyzed the data. NZ, JH and KJ drew the schematic graph. NZ drafted the manuscript. SC revised the manuscript. All authors listed have made a substantial, direct, and intellectual contribution to the work and approved it for publication.

## Funding

This work was supported by the Key Project of Agriculture in Fujian Province of China (No. 2021N0001, 2021NZ033016), and Fundamental Research Funds for the Central Universities (No. 20720200114).

## Acknowledgments

We thank technician Xinming Chen of Xiao Deng Fisheries Technology Co., LTD for his help in obtaining and transporting experimental fish. We thank Chenchen Shi (PhD candidate) of the College of Ocean and Earth sciences, Xiamen University for her assistance in collecting samples for ultrastructure observation. We thank Dr. Luming Yao and Dr. Caiming Wu of the College of Life sciences, Xiamen University for their help in analyzing the ultrastructure of the skin. We thank Dr. Yuting Zhang of the College of Geography and Oceanography, Minjiang University, for his comments on the manuscript revision. We thank Professor. Wanshu Hong of the College of Ocean and Earth sciences, Xiamen University, for his comments on the manuscript revision.

## Conflict of interest

The authors declare that the research was conducted in the absence of any commercial or financial relationships that could be construed as a potential conflict of interest.

## Publisher’s note

All claims expressed in this article are solely those of the authors and do not necessarily represent those of their affiliated organizations, or those of the publisher, the editors and the reviewers. Any product that may be evaluated in this article, or claim that may be made by its manufacturer, is not guaranteed or endorsed by the publisher.
